# Oral health related quality of life and the prevalence of ageusia and xerostomia in active and recovered COVID-19 Patients

**DOI:** 10.7717/peerj.14860

**Published:** 2023-03-06

**Authors:** Mahnoor K.M. Saleem, Abhishek Lal, Naseer Ahmed, Maria S. Abbasi, Fahim Vohra, Tariq Abduljabbar

**Affiliations:** 1 Prosthodontics Department, Altamash Institute of Dental Medicine, Karachi, Pakistan; 2 Prosthodontics Department, University Sains Malaysia, Kelantan, Malaysia; 3 Prosthetic Dental Sceinces, King Saud University, Riyadh, Saudi Arabia

**Keywords:** COVID 19, Xerostomia, Loss of taste, Quality of life, Recovery

## Abstract

**Background:**

Salivary disturbance is associated with patients who either have an active coronavirus disease 2019 (COVID-19) or have recovered from coronavirus infection along with loss of taste sensation. In addition, COVID-19 infection can drastically compromise quality of life of individuals.

**Objective:**

This study aimed to analyze xerostomia, ageusia and the oral health impact in coronavirus disease-19 patients utilizing the Xerostomia Inventory scale-(XI) and the Oral Health Impact Profile-14.

**Methods:**

In this cross-sectional survey-based study, data was collected from 301 patients who suffered and recovered from COVID-19. Using Google Forms, a questionnaire was developed and circulated amongst those who were infected and recovered from coronavirus infection. The Xerostomia Inventory (XI) and Oral Health Impact Profile-14 were used to assess the degree and quality of life. A paired T-test and Chi-square test were used to analyze the effect on xerostomia inventory scale-(XI) and OHIP-14 scale scores. A *p*-value of 0.05 was considered as statistically significant.

**Results:**

Among 301 participants, 54.8% were females. The prevalence of xerostomia in participants with active COVID-19 disease was 39.53% and after recovery 34.88%. The total OHIP-14 scores for patients in the active phase of infection was 12.09, while 12.68 in recovered patients. A significant difference was found between the mean scores of the xerostomia inventory scale-11 and OHIP-14 in active and recovered COVID patients.

**Conclusion:**

A higher prevalence of xerostomia was found in COVID-19 infected patients (39.53%) compared to recovered patients (34.88%). In addition, more than 70% reported aguesia. COVID-19 had a significantly higher compromising impact on oral function of active infected patients compared to recovered patients.

## Introduction

The recent coronavirus disease-19 (COVID-19) pandemic is the great challenge to the health care system and the general population. Along with the global health crisis, it has also brought an unprecedented socio-economic crisis, which continues to have a devastating effect  (Xie et al., 2020; Ali et al., 2020). Patients with COVID-19 present with mild flu-like condition leading to life-threatening multi-organ failure, lasting for up to two weeks, with high mortality among patients with co-morbidities  (Ahmed et al., 2020c; Ahmed et al., 2020a; Neto et al., 2020; Abbasi et al., 2020; Zhou et al., 2020).

The infected individuals primarily contract the virus from other infected humans by respiratory droplets produced by sneezing and coughing. Primary symptoms that are displayed by the infected patient include fever, dry cough, sore throat, shortness of breath, and myalgia  (Wang et al., 2020b). Furthermore, as the disease progresses, other symptoms which may develop include disturbed taste and smell sensations, along with gastric upset such as diarrhea and constipation (Ali et al., 2020; Neto et al., 2020; Abduljabbar et al., 2020). Literature suggests that the viral infection of the salivary gland cells may alter the salivary constituents and composition. Critical salivary compounds like proteins, enzymes and hormones play an important part in perception of taste. Therefore, it is likely that the ageusia associated in COVID-19 patients is the result of changes in salivary composition associated with the salivary gland infection (Abduljabbar et al., 2020). The mechanism of COVID-19 disease as presented in previous studies, includes an interaction with angiotensin-converting enzyme inhibitor 2 (ACE-2) receptors, which are present throughout the body including lungs, blood vessels, brain, and salivary glands (Sarfaraz et al., 2020). Additionally, tissues present in the body that express ACE-2 receptors are prone to invasion of this virus and may display various symptoms according to the system being affected. Since ACE-2 receptors are present in the salivary glands, there is a possibility that the coronavirus can invade the salivary glands and cause inflammatory reactions leading to acute and chronic sialadenitis and dry mouth (Wang et al., 2020a; Fantozzi et al., 2020; Freni et al., 2020).

Xerostomia is a condition whereby secretion or production of saliva is reduced causing compromised oral functions (Villa, Connell & Abati, 2014; Mulyani et al., 2019). It is associated with burning sensation, abnormal taste, dysarthria, dysphagia, dysgeusia, and halitosis, having a negative impact on the quality of life (Abduljabbar et al., 2020; Millsop, Wang & Fazel, 2017; Niklander et al., 2017). It occurs in 5.5% to 46% of the general world population with females being most affected (Anwar et al., 2022). It is also common in healthy individuals, although most commonly associated with many underlying systemic conditions. Increasing age along with polypharmacy are reported as risk markers for xerostomia especially inhaled anti-asthmatic drugs (Kishore et al., 2022). Other agents that contribute to xerostomia are caffeine, alcohol, tobacco, and carbonated beverages. COVID-19 infection along with xerostomia will compromise the oral health-related quality of life of patients and an improvement in quality of life for recovered individuals is expected. However, to our knowledge from indexed literature, there limited evidence related to oral health and quality of life of active and recovered COVID-19 patients.

Timely identification and management of COVID-19 induced dry mouth could prove to be beneficial for the already suffering patient to maintain their diet and health with improved quality of life. Therefore, this study aimed to investigate the possible correlation between coronavirus and Xerostomia and to determine the prevalence of ageusia and xerostomia in active and recovered COVID-19 patients. In addition, the impact of coronavirus on quality of life regarding oral health will also be assessed.

## Materials & Methods

### Study sample and ethical statement

In this cross sectional analytical study, using the convenience sampling method, adults who had an active COVID-19 and later on recovered from it were included. Children and adolescents along with those who were not infected with coronavirus were excluded. The sample size was calculated through Open-Epi software. Considering the minimum frequency of xerostomia to be 45.9%. The power of the test was 80. A confidence interval of 95%. The margin of error is 5%. The estimated sample size for this study was 350 participants. The information of the participants collected through the survey was kept anonymous and confidential. Participation was voluntary and all participants completed informed consent. This study was conducted under the approval of the Ethical and Review Committee of Altamash Institute of Dental Medicine. (AIDM/EC/08/2020/05).

Initially, a short message about whether to be tested positive for coronavirus or not was sent to 750 individuals. Out of 750 individuals, 400 reported to be tested positive for SARS-CoV-2, and 350 agreed to participate in this study.

### Questionnaire and data collection

A questionnaire was constructed and validated through face and content validation process, furthermore, the reliability of the questionnaire was assessed through intra-class correlation and the internal consistency of items assessed was 0.92. The questionnaire was sent through email, and their responses were recorded. Forty-nine incomplete forms were excluded. A total of 301 completed questionnaires were received and included in the study.

The questionnaire consisted of two sections. The first included the demography of the patients including age, gender, occupation, level of education and smoking habits, systemic diseases, and finally if any medicines were being taken. The second section was focused on whether the patients experienced xerostomia symptoms or not, and how their quality of life regarding oral health was affected during active infection and after they recovered from it, using validated measurement tools. Xerostomia and the oral health impact of the coronavirus was measured using the Xerostomia Inventory Scale (XI) (Thomson et al., 1999) and Oral Health Impact Profile-14 (OHIP-14) scale (Slade, 1997), both being self-administered. Both the Xerostomia Inventory Scale (XI) and Oral Health Impact Profile-14 (OHIP-14) scale are adapted in the present study in accordance with there published license. The Xerostomia Inventory scale (XI) comprised of a total of 11 questions, with each question having five options as follows: 1 = Never, 2 = Hardly ever, 3 = Occasionally, 4 = Fairly often, and 5 = Very often. The Oral Health Impact Profile-14 scale comprised of a total of 14 questions related to seven dimensions: Functional limitation, physical pain, psychological discomfort, physical disability, psychological disability, social disability, and handicap. Each question in the OHIP-14 scale has five options as follows: 0 = Never, 1 = Rarely, 2 = Sometimes, 3 = Repeatedly, and 4 = Always. In both of the scales used, options in each question have its own numerical value which was added at the end to obtain a total score for each participant. For the Xerostomia Inventory scale (XI), the minimum score was 11 and the maximum was 55, with higher scores depicting greater severity in oral dryness. In the OHIP-14 scale, the minimum score was 0 with 56 being the maximum, and a score above 10 indicated poor Quality of Life (QoL).

The questionnaire was composed of two parts, the first part consisted of Xerostomia Inventory and Oral Health Impact Profile scales questions which the patients were asked to fill when they had active coronavirus infection. The second part of the questionnaire also had similar questions of the Xerostomia Inventory and Oral Health Impact Profile scales but these were addressing symptoms after recovery from coronavirus infection. The total score for each of the two scales in both parts of the questionnaire was added up. The total scores of OHIP and XI scales during active coronavirus infection were compared with scores of OHIP and XI scales when participants had recovered from it.

### Statistical analysis

Descriptive statistics were performed to obtain, the frequency, percentages, mean scores, and standard deviations of the quantitative and qualitative variables in the study *i.e.,* age, gender, education status, drugs, smoking and systematic illness history, total score, questionnaire items, and domains. Paired *T*-test and Chi-square test were used to analyze the effect of mean xerostomia inventory (XI) and OHIP-14 scale scores on age, gender, and COVID-19 patients. A *p*-value of ≤ 0.05 was considered statistically significant.

## Results

A total of 301 COVID-19 patients participated in the study in two phases (infection and recovery). The response rate from participants was 86%. More than one-half (54.8%) of the participants were females. The majority of participants (126 (41.9%)) were undergraduates by education status and mostly students (130 (43.2%)) from occupation. More than two-thirds of participants were nonsmokers as far as habits are concerned. While 87 (28.9%) participants were taking some medicines, bronchodilators (6.89%) were the most common medicine used by the participants. Similarly, 81 (26.9%) participants were suffering from a systematic illness, and hypertension was the most common illness 52 (17.3%). Furthermore, 167 (55.48%) participants were younger than 30 years of age, 121 (40.19%) were from the age group of 31 to 60 years and 13 patients were older than 60 years of age as presented in Table 1.

**Table 1:** General and demographic and characteristics of study participants (*n* = 301).

**Variables**	**Factors**	***n***	**(%)**
Gender	Male	136	45.2
	Female	165	54.8
Age	18–30 years	167	55.5
	31–40 years	50	16.6
	41–50 years	42	14.0
	51–60 years	29	9.6
	Above 60 years	13	4.3
Education Status	Undergraduate	126	41.9
	Graduate	93	30.9
	Post-graduate	48	15.9
	Below Undergraduate	34	11.3
Occupation	Student	130	43.2
	Business	56	18.6
	Healthcare Professional	35	11.6
	Engineer	13	4.3
	Teacher	17	5.6
	Labor work	1	0.3
	Others	49	16.3
Do you smoke?	Yes	59	19.6
	No	242	80.4
How many cigarettes per day?	1	216	71.8
	2	37	12.3
	4	12	4.0
	6	15	5.0
	More than 6	21	8.6
Are you taking any medicines currently?	Yes	87	28.9
	No	214	71.1
Are you suffering from any other disease currently?	Yes	81	26.9
	No	220	73.1

**Figure 1:**
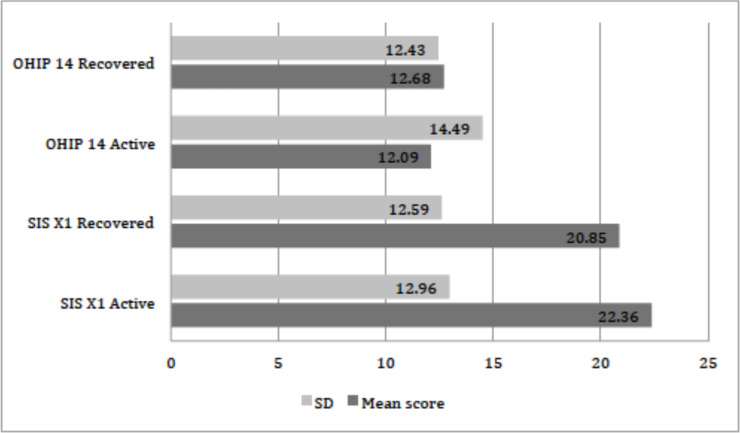
Distribution of the mean scores of OHIP-14 and SIS XI scale (*n* = 301). Figure is showing the mean scores of two scales used in this study, OHIP, Oral Health Impact Profile; SIS XI, Saliva Inventory Scale eleven; Active, patients with COVID-19 infection; Recovered, patients at healed stage of COVID-19 infection; SD, standard deviation.

The distribution of xerostomia inventory scale XI score for active and recovered COVID-19 patients is presented in Table 2. On the basis of scores 3, 4 and 5 from item 4 of the xerostomia inventory scale, the prevalence of xerostomia in participants with active COVID-19 disease was 39.53% and after recovery was 34.88%. The mean summative xerostomia inventory scale score was 22.36 ± 12.96 in active COVID-19 patients while in recovered patients 20.85 ± 12.59 as shown in Fig. 1. A significant difference was found between the mean scores of the xerostomia inventory scale among infected and recovered patients (paired *t*-test: *p* = 0.011). Additionally, a significant difference was also found between gender and xerostomia inventory scale scores in active (*p* = 0.002) and recovered COVID-19 patients (*p* = 0.001) (Chi-square test). Xerostomia was prevalent in young individuals between 18 to 30 years of age, with 17.60% in active while 15.61% in recovered cases. For older individuals of 60 years and above, xerostomia was in 4.98% and 4.31% active and recovered cases respectively. A significant difference was seen between the age of participants and xerostomia inventory scale scores in the active phase of COVID-19 infection (Chi-square test: *p* = 0.016). However, no significant difference (*p* = 0.473) was observed between age and recovered patient scores.

**Table 2:** Distribution of Saliva Inventory Scale in patients with COVID-19 (*n* = 301).

**Q**	**Saliva Inventory Scale**	**COVID-19 Patients**										
		**S**	**Never**		**Hardly ever**		**Occasionally**		**Fairly often**		**Very often**	
			*n*	%	*n*	%	*n*	%	*n*	%	*n*	%
1.	I sip liquids to help swallow food.	A	172	57.1	40	3.3	63	20.9	16	5.3	10	3.3
		R	185	61.5	51	16.9	44	14.6	13	4.3	8	2.7
2.	My mouth feels dry when eating a meal	A	174	57.8	30	10	53	17.6	30	10.0	14	4.7
		R	181	60.1	46	15.3	43	14.3	25	8.3	6	2.0
3.	I get up at night to drink.	A	140	46.5	42	14.0	56	18.6	44	14.6	19	6.3
		R	149	49.5	48	15.9	55	18.3	34	11.3	15	5.0
4.	My mouth feels dry	A	112	37.2	70	23.3	74	24.6	30	10.0	15	5.0
		R	120	39.9	76	25.2	66	21.9	26	8.6	13	4.3
5.	I have difficulty in eating dry foods	A	169	56.1	37	12.3	45	15.0	34	11.3	16	5.3
		R	173	57.5	51	16.9	46	15.3	22	7.3	9	3.0
6.	I suck sweets or cough lollies to relive dry mouth	A	193	64.1	40	13.3	35	11.6	27	9.0	6	2.0
		R	186	61.8	53	17.6	36	12.0	15	5.0	11	3.7
7.	I have difficulties swallowing certain foods	A	177	58.8	49	16.3	43	14.3	26	8.6	6	2.0
		R	179	59.5	42	14.0	45	15.0	26	8.6	9	3.0
8.	The skin of my face feels dry	A	162	53.8	44	14.6	42	14.0	24	8.0	14	4.7
		R	150	49.8	71	23.6	42	14.0	24	8.0	14	4.7
9.	My eyes feel dry	A	176	58.5	39	13.0	44	14.6	28	9.3	14	4.7
		R	174	57.8	58	19.3	37	12.3	15	5.0	17	5.6
10.	My lips feel dry	A	101	33.6	73	24.3	69	22.9	38	12.6	20	6.6
		R	105	34.9	86	28.6	64	21.3	28	9.3	18	6.0
11.	The inside of my nose feels dry	A	162	53.8	41	13.6	54	17.9	32	10.6	12	4.0
		R	174	57.8	39	13.0	49	16.3	29	9.6	10	3.3

**Notes.**

Q question Saliva Inventory Scale The top readings in each question are responses from active COVID-19 patients (A) while bottom readings are from COVID-19 recovered patients (R) *n* frequency % percentage COVID-19 coronavirus disease 2019 S patient status

The distribution of oral health impact profile scale 14 scores for active and recovered COVID-19 patients is described in Table 3. The total OHIP-14 score for patients in the active phase of infection was 12.09 ± 14.49, while 12.68 ± 12.43 in recovered patients as presented in Fig. 1. The prevalence of different domains in the OHIP-14 scale was as follows: Functional limitation was seen in 224 (74.41%) participants when assessed by scores 3 and 4 (Q1 and Q2). While physical pain was observed in 40 (13.28%) after summing up score 3 and 4 (Q3 and Q4). Psychological discomfort was found in 74 (24.58) (Q5 and Q6). Physical disability was seen in 81(26.91%) (Q7 and Q8). Psychological disability was found in 89 (29.56%) participants (Q9 and 10), whereas social disability (Q11, Q12) was evident in 86 (28.57%) participants. Being handicap was (Q13 and Q14) found in 77 (25.58%) subjects respectively (patients with active COVID-19 infection). Additionally, the highest individual OHIP-14 scale score was recorded for taste loss (70.09%) amongst the participants as described in Table 3.

**Table 3:** Distribution of OHIP scale in patients with COVID-19 (*n* = 301).

Q	OHIP scale	COVID-19 Patients										
		S	Never		Rarely		Sometimes		Repeatedly		Always	
			*n*	%	*n*	%	*n*	%	*n*	%	*n*	%
1.	Have you had trouble pronouncing any word?	A	174	57.8	47	15.6	67	22.3	11	3.7	2	0.7
		R	189	62.8	41	13.6	48	15.9	20	6.6	3	1.0
2.	Have you felt that your sense of taste has worsened?	A	24	8.0	15	5.0	51	16.9	163	54.2	48	15.9
		R	22	7.3	9	3.0	49	16.3	176	58.5	45	15.0
3.	Have you had painful aching in your mouth?	A	186	61.8	54	17.9	37	12.3	14	4.7	10	3.3
		R	182	60.5	61	20.3	35	11.6	16	5.3	7	2.3
4.	Have you found it uncomfortable to eat any foods?	A	184	61.1	44	14.6	57	18.9	9	3.0	7	2.3
		R	183	60.8	49	16.3	50	16.6	17	5.6	2	0.7
5.	Have you been self-conscious?	A	140	46.5	42	14.0	74	24.6	21	7.0	24	8.0
		R	154	51.2	37	12.3	75	24.9	15	5.0	20	6.6
6.	Have you felt tense?	A	103	34.2	51	16.9	73	24.3	50	16.6	24	8.0
		R	110	36.5	65	21.6	89	29.6	26	8.6	11	3.7
7.	Have your diet been unsatisfactory?	A	135	44.9	47	15.6	73	24.3	27	9.0	19	6.3
		R	152	50.5	42	14.0	75	24.9	18	6.0	14	4.7
8.	Have you had to interrupt meals?	A	144	47.8	58	19.3	64	21.3	26	8.6	9	3.0
		R	165	54.8	52	17.3	65	21.6	13	4.3	6	2.0
9.	Have you found it difficult to relax?	A	120	39.9	47	15.6	70	23.3	39	13.0	25	8.3
		R	121	40.2	72	23.9	69	22.9	23	7.6	16	5.3
10.	Have you been a bit embarrassed?	A	148	49.2	43	14.3	85	28.2	16	5.3	9	3.0
		R	156	51.8	50	16.6	70	23.3	13	4.3	12	4.0
11.	Have you been a bit irritable with other	A	127	42.2	46	15.3	79	26.2	32	10.6	17	5.6
		R	142	47.2	53	17.6	76	25.2	21	7.0	9	3.0
12.	Have you had difficulty doing your usual job?	A	146	48.5	42	14.0	76	15.2	27	9.0	10	3.3
		R	166	55.1	53	17.6	52	17.3	17	5.6	13	4.3
13.	Have you felt that life, in general, was less satisfying?	A	144	47.8	35	11.6	81	26.9	26	8.6	15	5.0
		R	159	52.8	39	13.0	58	19.3	30	10.0	15	5.0
14.	Have been totally unable to function?	A	166	55.1	40	13.3	59	19.6	26	8.6	10	3.3
		R	174	57.8	51	16.9	50	16.6	20	6.6	6	2.0

**Notes.**

Q question Q1 and Q2 functional limitation Q3 and Q4 physical pain Q5 and Q6 psychological discomfort Q7 and Q8 physical disability Q9 and Q10 psychological disability Q11 and Q12 social disability Q13 an 14 handicap OHIP Oral Health Impact Profile, The top readings in each question are responses from active COVID-19 patients (A) while bottom values are from COVID-19 recovered (R) patients *n* frequency % percentage, COVID-19:coronavirus disease 2019 S patient status

The domains of OHIP-14 scale in COVID-19 recovered patients were observed in the study. The functional limitation was compromised in 54 (17.94%) participants when assessed by scores 3 and 4 (Q1 and Q2), while physical pain was felt by 42 (13.95%) individuals, when summed up by score 3 and 4 (Q3 and Q4). Psychological discomfort was evident in 72 (23.92%) participants (item Q5 and Q6). Physical disability was seen in 51 (16.94%) subjects (Q7 and Q8) and psychological disability was found in 64 (21.26%) (Q9 and Q10). Social disability was apparent in 60 (19.93.%) participants (Q11 and Q12) and handicap was found in 71 (23.58%) patients (Q13 and Q14) respectively. Similarly, the highest individual OHIP-14 scale score was recorded for taste loss (73.42%) amongst the participants (sum of negative answers 3 and 4 in item number 2) as presented in Table 3. There was a significant difference (paired *t*-test: *p* = 0.001) between the mean scores of the OHIP-14 scale in active and recovered COVID-19 patients. Furthermore, a significant difference was also found between gender and OHIP-14 scale score in active COVID-19 patients (Chi-square tests: *p* = 0.002) and recovered patients (*p* = 0.003). The loss of taste was prevalent in young individuals 18 to 30 years of age (42.19%) while (10.29%). in older individuals 60 years and above. Moreover, there was no significant difference between the age of participants and OHIP-14 scores in active and recovered patients (Chi-square test: *p* = 0.076, *p* = 0.069) as shown in Table 4.

**Table 4:** Comparison of demographics with xerostomia and OHIP-14 scale mean scores in infected and recovered COVID-19 patients (*n* = 301).

Variable	Xerostomia inventory scale	
	Infected	Recovered
Gender	0.002^*^	0.001^*^
Age	0.016^*^	0.473
OHIP-14 scale		
Gender	0.002^*^	0.003^*^
Age	0.076	0.069

**Notes.**

* A *p*-value 0.05 was considered significant.

## Discussion

Currently, COVID-19 is one of the major threats to the health of human life. Its early detection is critical in prevention and management of the disease. In the present study, it was observed COVID-19 was more prevalent in females then and majority of the participants were younger than 30 years of age which is in contrast to a study by Ali et al. (2020) which reported that adult male patients with a median age between 34–59 years have a greater incidence of developing SARS-CoV-2 infection. Our findings are supported by the fact that 63% of population of the country where the data was collected (Pakistan) is comprised of youth aged individuals (Hafeez & Fasih, 2018). The present study also supports the findings of Berlin et al. (2020) who suggested that the published data on the association of COVID-19 with smoking status are only descriptive and conclusions from it cannot be drawn (Ahmed et al., 2020b). Furthermore, evidence suggests that underlying health conditions such as hypertension, diabetes, and coronary heart disease increase the risk of morbidity in COVID-19 patients (Abbasi et al., 2020; Wang et al., 2020a; Berlin et al., 2020). Nearly 27% of the participants in the present study suffered with systematic illness with hypertension being the most common. Studies by Zhou et al. (2020) and Wang et al. (2020a) are also in accordance to the findings by the current study as majority (48%) of patients had comorbidity, with hypertension being the most common followed by diabetes (Abbasi et al., 2020).

Furthermore, this study reported on the prevalence of xerostomia in patients with active COVID-19 disease, which was 39.53% and after recovery 34.88%. Earlier studies have also reported that COVID-19 patients experiences xerostomia though the temporal sequence of xerostomia and COVID-19 diagnosis is not clear and warrants further exploration (Abrar et al., 2021; Anzar et al., 2021; Rodríguez, Romera & Villarroel, 2020). In addition, xerostomia is known to be related to a wide range of viral infections (Nasiri, 2020) and SARS-CoV-1 has been shown to infect epithelial cells in salivary gland ducts in rhesus macaques  (Nigar et al., 2022). Moreover, Freni et al. (2020) reported xerostomia in COVID-19 patients with a statistically significant difference between active and recovered patients (Fantozzi et al., 2020). This is similar to the observations in the present study, as a significant difference was found between the mean scores of xerostomia inventory scale between active and recovered patients. Additionally, a significant difference was also found between gender and xerostomia inventory scale scores in active and recovered COVID-19 patients with the female having more prevalence. This corresponds to the study by Biadsee et al. (2020), where 56.2% experienced xerostomia with females having more prevalence (61%).

Xerostomia is multifactorial and is commonly associated with old age and polypharmacy (Mulyani et al., 2019; Nigar et al., 2022). Other factors include emotional stress and anxiety disorders, along with agents like caffeine, alcohol, tobacco, and carbonated beverages (Ahmed et al., 2020c). Many of these contributing factors are also associated with COVID-19 patients, so the exact cause of xerostomia in COVID-19 is yet to be discovered, either it is due to the mentioned conditions or due to viral infection of salivary glands impeding the salivary flow (Mulyani et al., 2019; Nasiri, 2020; Ahmed et al., 2020c; Fathi et al., 2021). As all the above-mentioned studies are observational and the literature lacks objective evidence, therefore, it is recommended that objective evaluation with validated, repeatable and standardized tests to establish the frequency, extent, and cause of xerostomia in COVID-19 patients are performed.

COVID-19, affects the oral health-related quality of life of patients and OHIP-14 score among patients in the present study with active infection and recovery were 12.09 ± 14.49 and 12.68 ± 12.43 respectively. This is in line with the study by Niklander et al. (2017) with OHIP-14 scores among COVID-19 patients to be 20.1 ± 14.32 (Millsop, Wang & Fazel, 2017). A high prevalence of functional limitation was seen in participants of present study, which includes difficulty in speech and loss/altered taste. These findings are in accordance with a study by Vaira et al. (2020) in which majority of the patients reported gustatory disorders during SARS-CoV-2 infection (Chen et al., 2020). Similar findings are also observed in other studies (Mohamud et al., 2020; Giacomelli et al., 2020). Moreover, a smaller number of patients also complained of a painful mouth and discomfort in eating food which is also supported by Lechien et al. (2020) who reported loss of appetite and dysphagia amongst COVID-19 patients (Wee et al., 2020).

It is also established that COVID-19 is associated with psychiatric implications including delirium, depression, anxiety, and insomnia  (Holmes et al., 2020). Other coronaviruses also induce psychopathological sequelae through direct viral infection of the central nervous system (CNS) or indirectly *via* an immune response (Holmes et al., 2020; Wu et al., 2020). Moreover, psychological stressors such as social isolation, the psychological impact of a novel severe and potentially fatal illness, concerns about infecting others, and stigma are implicated in COVID-19 related psychiatric manifestation (Mazza et al., 2020). In the present study as well, psychological and social disability along with handicap symptoms were observed in more than a quarter of subjects.

The study showed that xerostomia and ageusia are associated with COVID-19 infections and quality of life including difficulty in eating, speech difficulty, social and psychological disorders are augmented in infection. Therefore, it is important that a detailed and comprehensive intraoral assessment should be performed in patients that were diagnosed with COVID-19 in order to establish any oral manifestation that might be related. Moreover, the dentist should improve the examination of salivary glands and saliva flow in order to perform early diagnoses related to changes in the glandular parenchyma that might be affected by the virus.

The study has some limitations. First, the sample size is small and includes mild-moderate patients, thus cannot represent general COVID-19 patients. Some of the COVID-19 survivors that we were able to reach did not want to participate in the study. In addition, the salivary flow before the infection and other factors like stress, that might have affected the oral health could not have been evaluated. The occurrence of xerostomia is a possible outcome of the viral infection in the patients observed, however it should also be associated with treatment of the infection. In addition, it may be essential to carry out the measurement of the salivary flow before and after the COVID-19 diagnosis to demonstrate a close correlation of it with the virus. The study targeted only COVID-19 patients. There may be a bias because people who have xerostomia or any oral problem would intend to reply to the questionnaire. Healthy people who do not have any oral problems would not want to participate in this study.

## Conclusions

Both active and recovered COVID-19 patients reported decreased salivation and altered taste sensation but much higher prevalence was reported in infected ones. Similarly, oral health impact profile was much more compromised in active infected patients compared to recovered patients.

##  Supplemental Information

10.7717/peerj.14860/supp-1 Supplemental Information 1 DATA of study SPSS

## Additional Information and Declarations

### Data Availability

The following information was supplied regarding data availability: The raw data is available in the Supplemental Files.
